# An *Arabidopsis* E3 ligase *HUB2* increases histone H2B monoubiquitination and enhances drought tolerance in transgenic cotton

**DOI:** 10.1111/pbi.12998

**Published:** 2018-09-04

**Authors:** Hong Chen, Hao Feng, Xueyan Zhang, Chaojun Zhang, Tao Wang, Jiangli Dong

**Affiliations:** ^1^ State Key Laboratory of Agrobiotechnology College of Biological Sciences China Agricultural University Beijing China; ^2^ Key Laboratory of Cotton Genetic Improvement Ministry of Agriculture Cotton Research Institute Chinese Academy of Agriculture Sciences Anyang China

**Keywords:** *Gossypium hirsutum* Linn., *HISTONE MONOUBIQUITINATION 2 (HUB2)*, drought, transgenic plants, histone monoubiquitination, histone methylation

## Abstract

The *HUB2* gene encoding histone H2B monoubiquitination E3 ligase is involved in seed dormancy, flowering timing, defence response and salt stress regulation in *Arabidopsis thaliana*. In this study, we used the cauliflower mosaic virus (CaMV) 35S promoter to drive *AtHUB2* overexpression in cotton and found that it can significantly improve the agricultural traits of transgenic cotton plants under drought stress conditions, including increasing the fruit branch number, boll number, and boll‐setting rate and decreasing the boll abscission rate. In addition, survival and soluble sugar, proline and leaf relative water contents were increased in transgenic cotton plants after drought stress treatment. In contrast, RNAi knockdown of *GhHUB2* genes reduced the drought resistance of transgenic cotton plants. *AtHUB2* overexpression increased the global H2B monoubiquitination (H2Bub1) level through a direct interaction with GhH2B1 and up‐regulated the expression of drought‐related genes in transgenic cotton plants. Furthermore, we found a significant increase in H3K4me3 at the *DREB* locus in transgenic cotton, although no change in H3K4me3 was identified at the global level. These results demonstrated that *AtHUB2* overexpression changed H2Bub1 and H3K4me3 levels at the *GhDREB* chromatin locus, leading the *GhDREB* gene to respond quickly to drought stress to improve transgenic cotton drought resistance, but had no influence on transgenic cotton development under normal growth conditions. Our findings also provide a useful route for breeding drought‐resistant transgenic plants.

## Introduction

As the global temperature has increased by approximately 0.13 °C per decade since 1950 and rainfall has decreased, drought is becoming one of the most serious threats to agricultural productivity worldwide (Lobell *et al*., 2011). Ten percent of the arable land globally has been affected by desertification, and plants growing in such unsuitable environments are unable to reach their full genetic potential, exhibiting reduced growth and a more than 50% decrease in production (Bartels and Sunkar, 2005; Zhu, 2002). Cotton (*Gossypium hirsutum*) is an important commercial crop, as its fibre is used in many textiles and its seeds are rich in protein and produce edible cottonseed oil. Cotton is mainly distributed in hot and dry regions such as Xinjiang Province in China, South Africa, and Central Asia, which makes cotton more tolerant to drought stress than other crops; however, drought is still one of the most restrictive factors for its production and fibre quality (Soth, 1999). Understanding the basic molecular mechanisms of drought stress and using genetic engineering to develop more productive stress‐tolerant crops will allow us to better address this tremendous challenge.

Traditional breeding has made some progress in developing plants that can cope with abiotic stress, but this ability is often transferred along with other undesirable traits, and reproductive barriers between species make it difficult to obtain desired traits quickly and effectively (Varshney *et al*., 2011). Studies of the molecular mechanisms by which plants respond to abiotic stress have already identified a series of genes involved in stress tolerance; these genes encode various kinds of proteins, including transcription factors (TFs), enzymes, molecular chaperones and other functional proteins. These findings have provided a solid foundation for genetic breeding and have helped accelerate breeding speed (Umezawa *et al*., 2006). Individual or combined expression of the *Arabidopsis* H^+^‐pyrophosphatase gene *AtAVP1* and the vacuolar Na^+^/H^+^ antiporter gene *AtNHX1* in cotton reduces the cell water potential and promotes the development of the root system to increase drought and salt tolerance (Pasapula *et al*., 2011; Shen *et al*., 2015). The expression of some genes involved in abscisic acid (ABA) biosynthesis (*LOS5/ABA3*) or signal transduction (*ABI3* and *ABI5*) can enhance the activity of ABA‐dependent pathways to increase transgenic cotton drought tolerance and productivity (Mittal *et al*., 2014; Yue *et al*., 2012). Other genes encoding functional proteins can also be transformed into cotton to improve drought tolerance and agronomic traits (Divya *et al*., 2010; Parkhi *et al*., 2009; Yu *et al*., 2016; Zhang *et al*., 2014). Although genetic breeding has made some progress through the expression of several candidate abiotic stress response genes in cotton, the identification of new drought resistance genes would enrich our germplasm resources and deepen our understanding of drought‐resistant breeding.

The N‐termini of histones can be covalently modified by methylation, acetylation, phosphorylation, ADP ribosylation, biotinylation and monoubiquitination. Unlike polyubiquitination, which marks proteins for proteasome‐mediated degradation, monoubiquitination alters the subcellular localization or the biochemical or molecular functions of target proteins (Deshaies and Joazeiro, 2009). Histone monoubiquitination, especially H2B monoubiquitination (H2Bub1), is always associated with histone H3K4 or H3K36 methylation and cooperatively regulates gene expression. In the *Arabidopsis* genome, there are two C3HC4 RING‐type E3 ligases, *HISTONE MONOUBIQUITINATION1* (*HUB1*) and *HUB2*, which can form heterotetramer catalyse histone H2B monoubiquitination in cooperation with the E2 ubiquitin‐conjugating enzyme (UBC1 and UBC2), exerting multiple effects on plant growth and development (Cao *et al*., 2008; Xu *et al*., 2009). *Arabidopsis hub1* and *hub2* mutants exhibit a global lack of H2Bub1, and this deficiency is associated with dysregulation of key regulators of the cell cycle G2‐to‐M transition and changes in the expression of seed dormancy‐related genes (Fleury *et al*., 2007; Liu *et al*., 2007). Deficiencies in H2Bub1 modification can also affect histone H3K4me3 and H3K36me3 levels, which modulate the expression of the floral repressor *FLOWER LOCUS C (FLC)* to regulate flowering time (Cao *et al*., 2008; Gu *et al*., 2009). Recent studies have shown that H2Bub1 affects photomorphogenesis, cutin and wax composition and the circadian clock through regulating the expression of related genes (Bourbousse *et al*., 2012; Himanen *et al*., 2012; Menard *et al*., 2014). In addition, HUB1 and HUB2 facilitate plant biotic stress responses through directly monoubiquitinating histone H2B at the *R* gene locus (Zou *et al*., 2014) and other defence‐related gene loci (Dhawan *et al*., 2009; Hu *et al*., 2014). In addition, its homologs in rice (*Oryza sativa*) can participate in late anther development through direct functions related to tapetum degradation‐related genes (Cao *et al*., 2015). *AtHUB2* can also be induced by salt or mannitol and participates in salt stress regulation (Zhou *et al*., 2017).

As a range of cellular processes are regulated by *HUB2*, we ectopically expressed *AtHUB2* in the cotton cultivar CCRI24 and found that *AtHUB2* transgenic cotton plants exhibited significantly elevated drought tolerance under both greenhouse and field conditions. Using immunoblotting and Chromosome ImmunoPrecipitation (ChIP), we found that *AtHUB2* transgenic cotton has a higher global level of H2Bub1, which led to an increase in H3K4me3 at the *GhDREB* gene, quickly triggering *GhDREB* expression under drought stress conditions. This work is the first report that *AtHUB2* participates in the drought stress response and that heterologous expression of *AtHUB2* improves drought resistance in transgenic cotton through influencing histone modifications.

## Results

### *AtHUB2* plays a positive role in *Arabidopsis* under drought stress conditions

**Figure 1:**
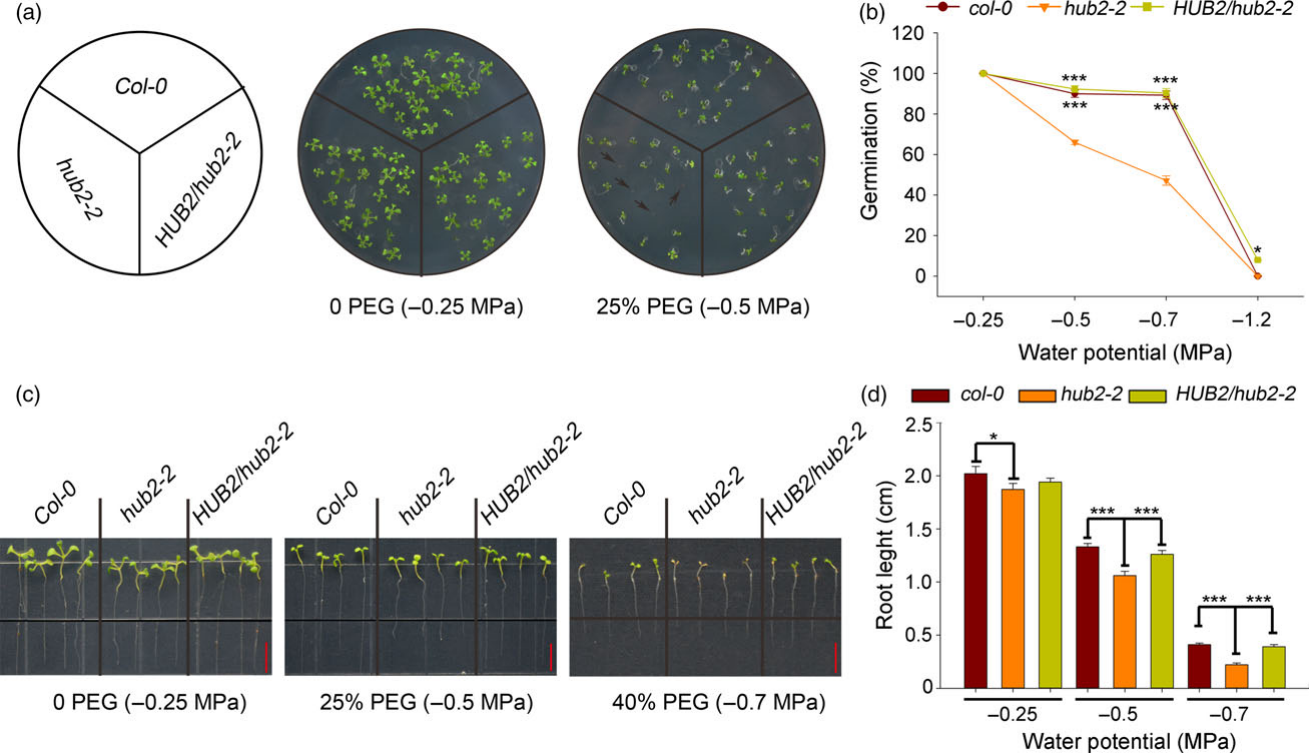
Response of *Athub2* mutants to osmotic stress. (a) Seeds of the indicated genotypes were germinated on 1/2 MS medium or 1/2 MS previously infused with 25% PEG. Photographs were taken 7 days after germination. Black arrows indicate un‐germinated seeds. (b) Percentages of seeds that germinated in different concentrations of PEG. (c) Root growth of the indicated genotypes. Six‐day‐old seedlings were transferred from 1/2 MS to 1/2 MS medium previously infused with different concentrations of PEG. Photographs were taken 4 and 7 days after transfer, bar = 0.5 cm. (d) Quantification of relative root length in (c). Vertical bars represented standard deviation (SD). (*n* ≥ 30, *P < 0.05; ***P < 0.001).

*AtHUB2* is a pleiotropic gene that participates in many aspects of plant growth, developmental regulation and defence or immune response modulation. However, studies on the potential role of this gene in abiotic stress regulation remain limited, with the exception of one report showing that H2B monoubiquitination is involved in salt stress regulation (Zhou *et al*., 2017). To test whether *AtHUB2* functions under drought stress in *Arabidopsis*, we analysed seedlings of the *Arabidopsis* Col‐0 (WT) line, the *hub2* mutant line *hub2‐2* and a complemented *hub2‐2* mutant line (*HUB2/hub2‐2*) to determine their tolerance to osmotic and drought stress. Seeds of *hub2‐2*,* HUB2/hub2‐2* and WT plants were grown in media containing different concentrations of polyethylene glycol 8000 (PEG‐8000). As the PEG‐8000 concentration increased from −0.25 to −0.7 MPa, the germination rates of *hub2‐2, HUB2/hub2‐2* and WT seeds all decreased, but the germination rates of WT and *HUB2/hub2‐2* seeds were significantly higher than that of *hub2‐2* seeds. In addition, in medium without PEG‐8000, *hub2‐2*,* HUB2/hub2‐2* and WT seeds all germinated (Figure 1a,b). Six‐day‐old normally growing WT, *HUB2/hub2‐2* and *hub2‐2* seedlings were transferred to media containing different concentrations of PEG‐8000. After 6 days of treatment, root growth was measured with ImageJ (NIH, USA) software, and the root growth of *hub2‐2* mutants was found to be significantly inhibited compared with that of WT and *HUB2/hub2‐2* plants. When osmotic stress reached −0.7 MPa, WT and *HUB2/hub2‐2* seedlings had more green leaves than *hub2* mutants. Under normal growth conditions, there were no differences in root length between WT and *HUB2/hub2‐2,* but their roots were slightly longer than those of *hub2‐2* plants (Figure 1c,d).

Furthermore, soil‐grown *hub2‐2* mutants were more sensitive to drought stress than WT and *HUB2/hub2‐2* plants. Plants were allowed to germinate and grow normally for 14 days and were then subjected to drought treatment for 12 days and re‐watering for 3 days, and their survival rates were then recorded (Figure S1a). The survival rate of the WT plants was 91.94%, and that of the *HUB2/hub2‐2* seedlings was 85.17%, while only 64.15% of the *hub2‐2* mutants survived. The survival rates of the WT and *HUB2/hub2‐2* plants were significantly higher than that of the *hub2‐2* mutants (Figure S1b). We next examined the water loss rate of leaves by measuring the weight of the leaves at different drought time points. The *hub2‐2* mutants lost water faster than the WT and *HUB2/hub2‐2* plants, while there was no difference in the water loss rate between the WT and *HUB2/hub2‐2* leaves (Figure S1c). These results demonstrated that *AtHUB2* plays a positive role in the *Arabidopsis* drought stress response.

### *AtHUB2* overexpression improves the agricultural traits of transgenic cotton grown in a waterproof shed

Since *AtHUB2* was found to be involved in drought stress responses, we generated transgenic cotton overexpressing *AtHUB2* to test the effects on drought stress resistance. *AtHUB2* has four alternative splicing isoforms in which key functional sites are conserved (Figure S2). To generate *AtHUB2*‐overexpressing transgenic cotton, the 1152 bp full‐length coding sequence (CDS) of one of *AtHUB2* isoforms, At1g55250.2, with Myc tag added to its 5′ end was cloned into the pCAMBIA1305.1 vector under the control of the cauliflower mosaic virus (CaMV) 35S promoter. The pCAMBIA1305.1‐AtHUB2 construct was introduced into the cotton cultivar CCRI24 via *Agrobacterium*‐mediated transformation, and 12 independent transgenic lines (T0) were produced. We then used the T0 plants to raise T1 progeny via self‐fertilization, and the presence and integrity of the transgene were confirmed by PCR analysis of genomic DNA with primers specific for the CaMV 35S promoter and *AtHUB2* (Figure S3a). Three independent homozygous T2 transgenic plants, line2, line4 and line6, were further identified by Southern blotting analysis with single‐ or two‐copy inserts (Figure S3b). Semi‐quantitative RT‐PCR and quantitative real‐time PCR (RT‐qPCR) showed that the three transgenic lines exhibited similar transcription levels (Figure S3c,d), and immunoblotting analyses with an anti‐Myc antibody showed that the AtHUB2 protein could be detected only in the transgenic lines (Figure S3e). These results showed that *AtHUB2* was successfully transformed into the cotton cultivar CCRI24 and could be inherited in the offspring.

**Figure 2:**
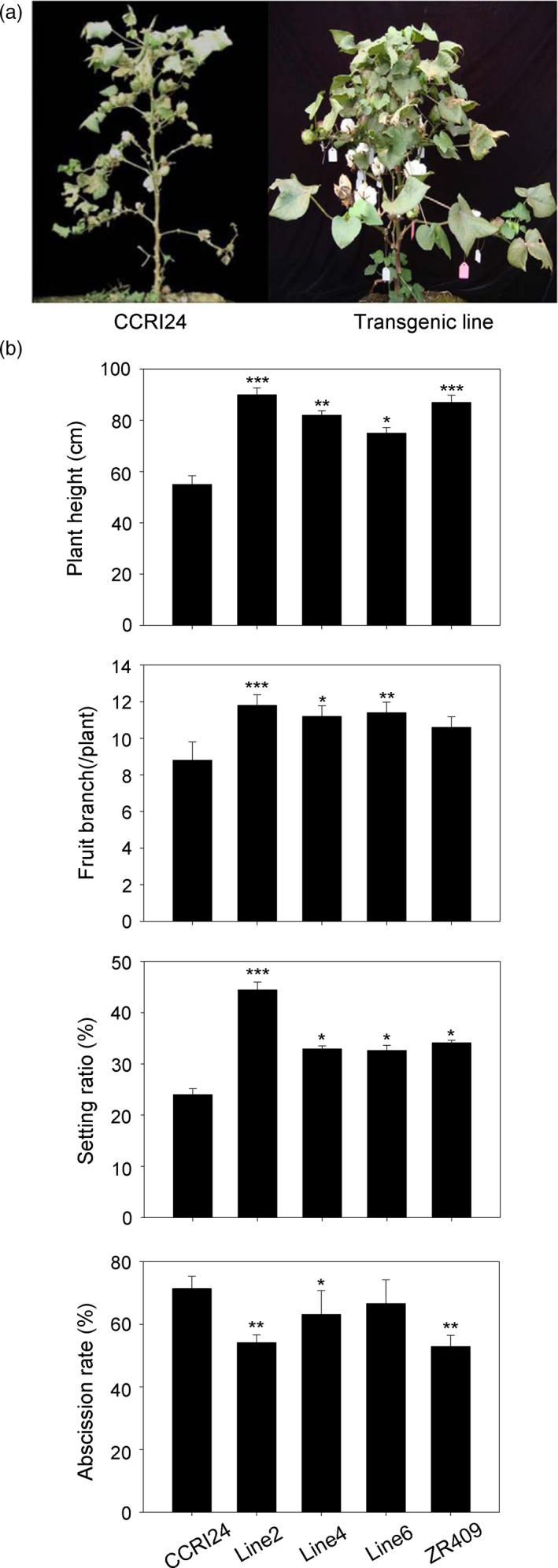
Agronomic traits of different lines grown in a waterproof shed. (a) Drought tolerance assay in the field under a waterproof shed in Anyang, Henan province, China. The photographs were taken 1 month after re‐watering. (b) Plant height, fruit branching, boll‐setting rates and abscission rates of controls (transgenic acceptor CCRI24 and drought‐resistant ZR409) and *AtHUB2*‐expressing cotton lines (line2, line4 and line6) in the field under drought stress conditions (*n* ≥ 30). Vertical bars represented SD. (*P < 0.05; **P < 0.01; ***P < 0.001).

Phenotypic experiments were carried out at the Institute of Cotton Research of the Chinese Academy of Agricultural Science (CAAS), Anyang, Henan province. Three *AtHUB2* transgenic lines (line2, line4 and line6), the control cultivar CCRI24 and the drought‐resistant control line ZR409 were grown on test land. Statistical analysis of agricultural characteristics indicated that under normal growth conditions, there were no obvious developmental differences between the transgenic and control plants, except that line6 had a lower plant height (Figure S4). However, when the plants were grown under drought conditions, as shown in Figure 2, the plant height, fruit branching and boll‐setting rate were significantly increased in all three transgenic lines. Moreover, compared with the control plants, line2 in particular exhibited remarkable increases and presented greener and less wilted leaves (Table S1). In addition, with the exception of line6, the abscission rate was significantly reduced in the transgenic lines compared with the control plants. These results suggest that *AtHUB2* overexpression did not affect the agricultural traits of transgenic cotton under normal growth conditions but could improve these traits under drought stress conditions.

### *AtHUB2* overexpression in cotton enhances drought tolerance and regulates drought stress‐related gene expression in the greenhouse

**Figure 3:**
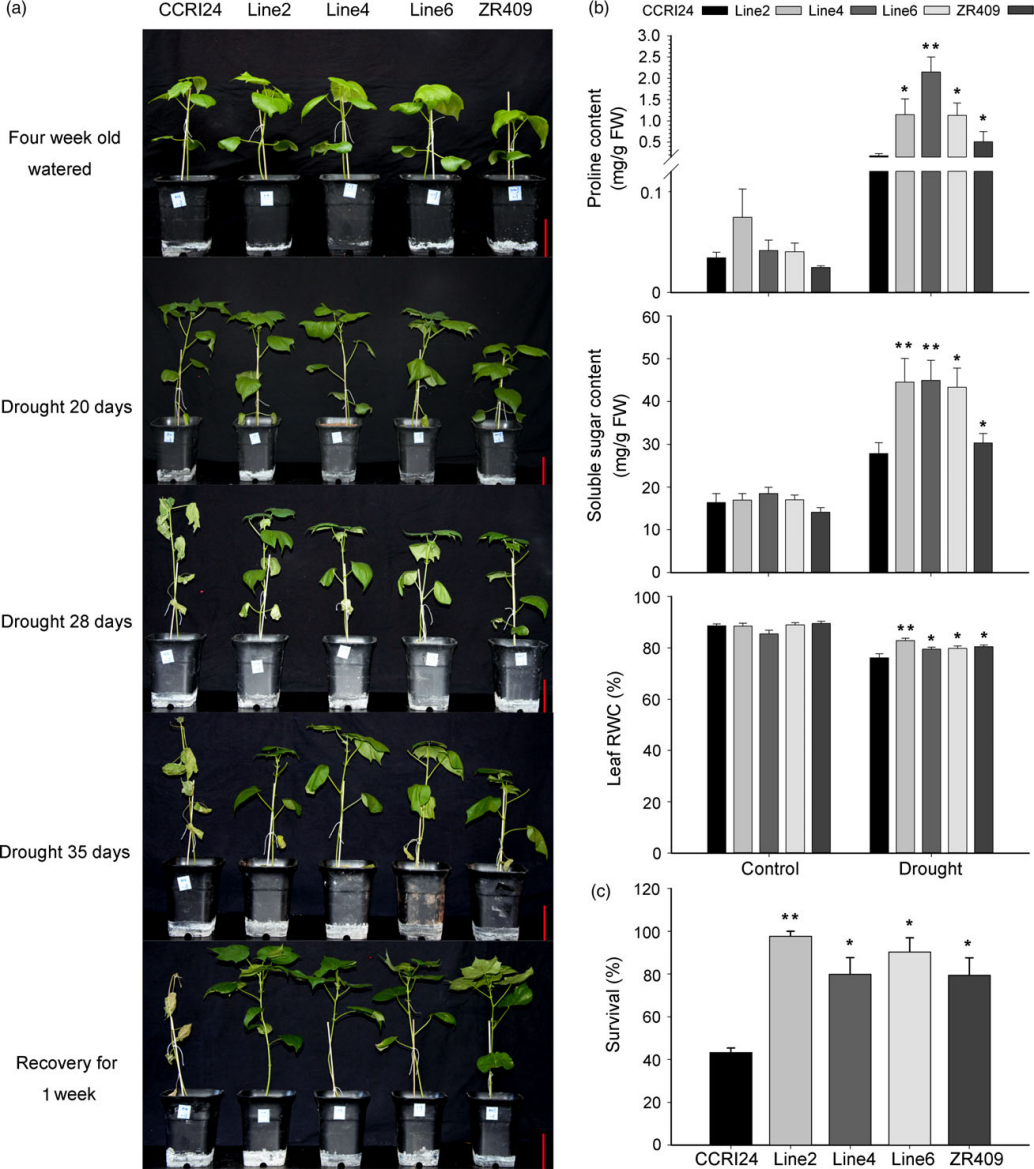
*AtHUB2* significantly enhances the drought tolerance of transgenic cotton in the greenhouse. (a) Drought tolerance of control plants and *AtHUB2*‐expressing cotton plants. Photographs were taken at 4 weeks after germination, after 20, 28 and 35 days of drought stress, and after a 1‐week recovery period. Bar = 7.5 cm. (b) Leaf relative water content (RWC), proline content and soluble sugar content of transgenic cotton and control plants with or without drought treatment (*n* ≥ 30). (c) Survival rates of plants after 1 week of recovery (*n* ≥ 30). Vertical bars represent SD. (*P < 0.05; **P < 0.01).

To further confirm that *AtHUB2* overexpression can improve the drought resistance of transgenic cotton, we performed a drought tolerance assay in the greenhouse. The three transgenic lines described above as well as the controls were grown under controlled irrigation conditions and then subjected to drought stress 4 weeks after germination. As shown in Figure 3a, no obvious difference was observed between the transgenic and control plants under normal growth conditions, but after 20 and 28 days of drought stress, the control leaves were much more wilted than those of the transgenic lines. After 35 days of drought treatment, the leaves of CCRI24 plants were essentially withered, while those of the transgenic plants were still green and exhibited only slight wilting. After 1 week of recovery, most of the transgenic plants had recovered, showing survival rates from 80% to 97.78%, while the CCRI24 cultivar had a survival rate of only 47.74% (Figures 3c and S5).

We then measured and compared soluble sugar content, proline content and leaf relative water content (RWC) between the controls and transgenic cotton plants with or without drought treatment (Figure 3b). Without drought treatment, the soluble sugar and proline contents were low, and no differences were observed among CCRI24 and ZR409. During drought treatment, the proline and soluble sugar contents increased significantly in both transgenic and control plants, but the increases in transgenic plants were more significant than those in control plants. In addition, the leaf RWC decreased after drought treatment in both control and transgenic plants but was still significantly higher in transgenic lines than in control plants.

**Figure 4:**
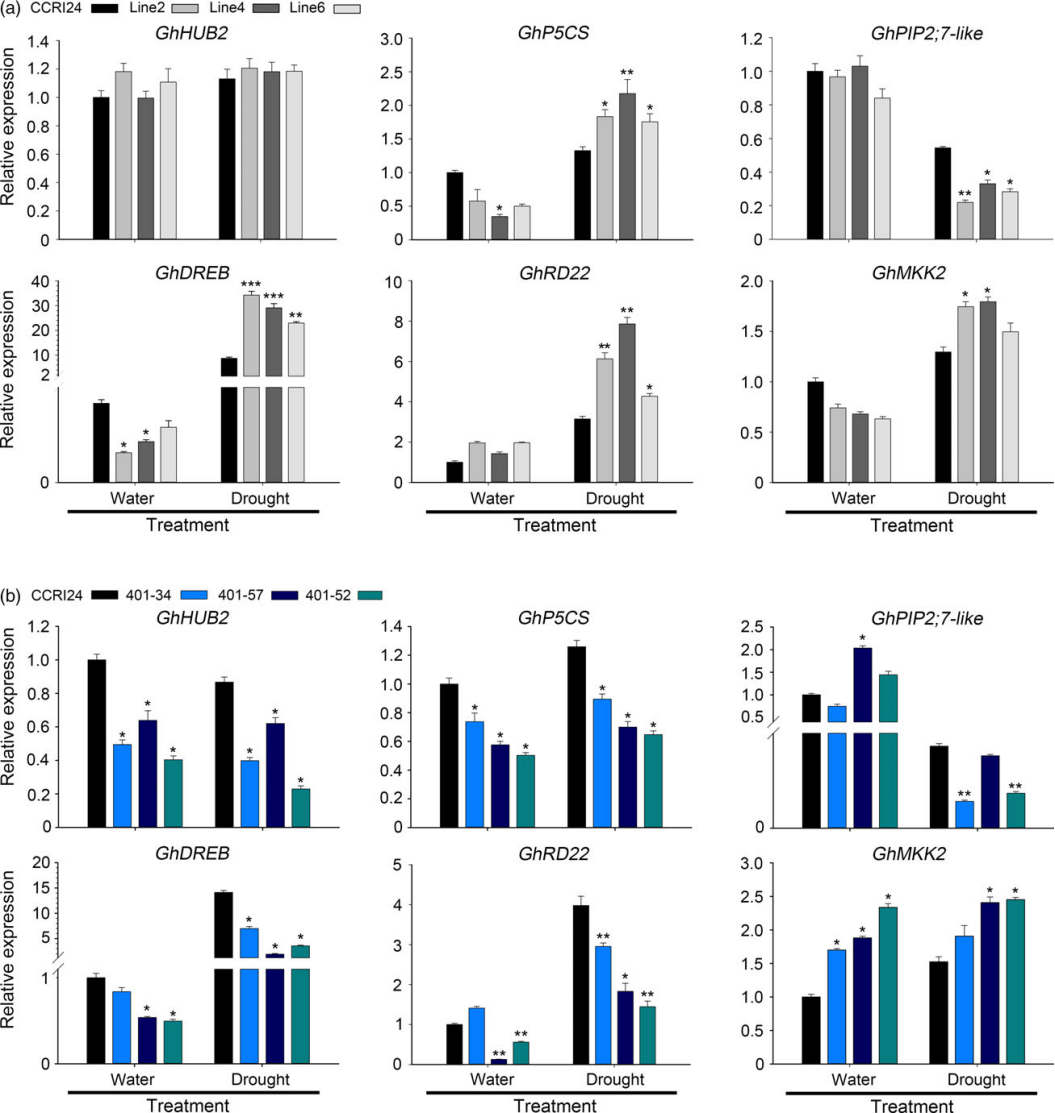
RT‐qPCR analyses of the expression of stress‐responsive genes in control, *AtHUB2* overexpression lines (a) and *GhHUB2‐*knockdown lines (b). The RNA extracted from the leaves of the indicated genotypes (4 weeks old) before or after air‐drying for 4 h. Values determined via RT‐qPCR were normalized to the expression of *GhUBI1*. Vertical bars represent the SD. (*P < 0.05; **P < 0.01, ***P < 0.001).

We next detected the expression of drought stress‐related genes. Without drought treatment, the expression levels of the ABA pathway‐dependent gene *responsive to dehydration (GhRD22)*, the ABA pathway‐independent gene *dehydration‐responsive element‐binding protein* (*GhDREB*), the proline synthesis‐related gene δ*1‐pyrroline‐5‐carboxylate synthetase* (*GhP5CS*)*,* the homologue of the rice negative drought stress regulation factor *plasma membrane intrinsic protein 2;7* (*OsPIP2;7*) in cotton *GhPIP2;7‐like* (Ning *et al*., 2011) and *mitogen‐activated protein kinase kinase 2* (*GhMKK2*) were not significantly different between the transgenic plants and control plants. After air‐drying for 4 h, the expression levels of *GhRD22*,* GhDREB*,* GhP5CS* and *GhMKK2* were all up‐regulated in both the transgenic cotton and control plants, but the expression levels were significantly increased in the transgenic lines compared with the control line. In contrast, the expression of *GhPIP2; 7‐like* was significantly decreased in the transgenic lines compared with that in the control cotton (Figure 4a). Taken together, these results indicated that *AtHUB2* overexpression can significantly improve the drought tolerance of transgenic cotton and influence drought‐related gene expression.

### *GhHUB2*‐specific RNAi reduces drought tolerance in transgenic cotton

**Figure 5:**
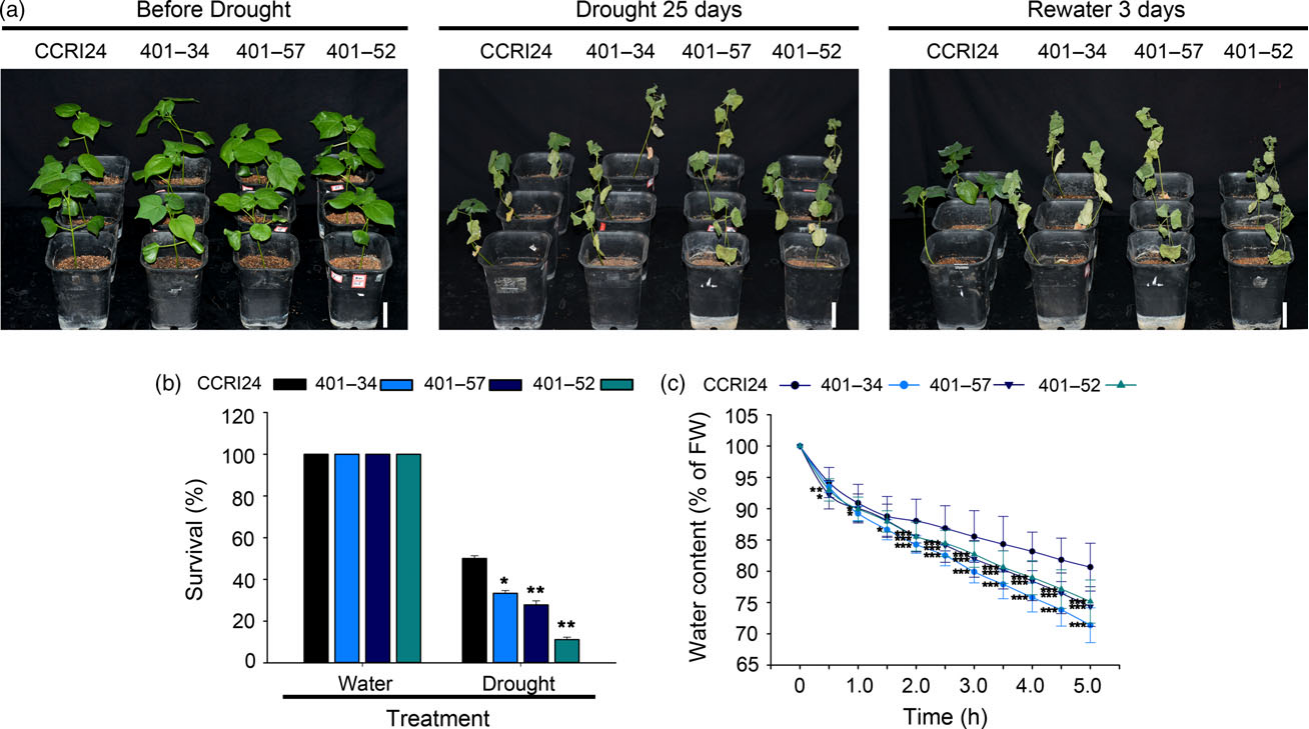
Response of *GhHUB2‐*knockdown lines to drought stress. (a) Drought tolerance of control CCRI24 and *GhHUB2‐*knockdown cotton plants. Photographs were taken at 4 weeks after germination, after 25 days of drought stress and re‐watering for 3 days. Bar = 7.5 cm. (b) Survival rates of plants after 25 days of drought stress and 3 days of recovery (*n* ≥ 30). (c) The volume of water lost from detached leaves of the indicated genotypes (4 weeks old) via air‐drying was measured as the percentage of change in the fresh weight (FW) of the leaves (*n* ≥ 30). Vertical bars represent SD. (*P < 0.05; **P < 0.01; ***P < 0.001).

To verify that the activity of *AtHUB2* is the cause of the drought effect observed in transgenic cotton, we used *GhHUB2*‐specific RNA interference (RNAi) transgenic plants to perform drought tolerance assays. *GhHUB2* RNAi transgenic cotton plants were generated previously in our lab. Two candidate *AtHUB2* homologs, *GhHUB2‐A* and *GhHUB2‐B*, were identified in *G. hirsutum* through multiple sequence alignment against the TM‐1 genome using the AtHUB2 protein sequence. The multiple sequence alignment results showed that these two GhHUB2s share 98.6% nucleotide identity and 98.4% amino acid sequence identity and that both contain a conserved C3HC4 RING finger domain in the C‐terminal region. Therefore, the 272 bp region from the 3′ end, which included the RING domain shared by both GhHUB2s, was chosen for RNAi (Feng *et al*., 2018). Three T4 generation plants with significantly down‐regulated expression of *GhHUB2* were used to perform drought tolerance assays (401‐34, 401‐57 and 401‐52). Under normal growth conditions, there were no developmental differences between WT and *GhHUB2*‐knockdown plants. However, after drought treatment, the *GhHUB2‐*knockdown plants exhibited more wilting than the WT plants (Figure 5a). While 50% of WT plants survived, only 11.11%–33.33% of *GhHUB2‐*knockdown plants survived after 25 days drought treatment and 3 days of recovery (Figure 5b). Moreover, the water loss rates of leaves detached from *GhHUB2‐*knockdown plants were higher than those of leaves from WT plants (Figure 5c). To confirm that the reduced drought resistance was due to down‐regulation of *GhHUB2* expression, RT‐qPCR was performed to evaluate the transcription level of the *GhHUB2* gene using template RNAs derived from the leaves of cotton plants exhibiting normal growth or exposed to air drought for 4 h. The results showed that a reduction in the expression of *GhHUB2* to 40.35%–62.01% of WT expression was evident. The expression of *GhHUB2* was not affected by drought in WT, *GhHUB2‐*knockdown plants or even *AtHUB2*‐overexpressing plants (Figure 4). Then, we examined the expression of drought‐related genes in *GhHUB2‐*knockdown and WT plants. As shown in Figure 4b, the expression levels of *GhDREB, GhP5CS* and *GhRD22* were decreased compared with those in WT plants under normal growth conditions and were then up‐regulated by drought stress, whereas the expression levels of these genes were still significantly lower in *GhHUB2‐*knockdown plants than in WT plants. The expression levels of *GhPIP2; 7‐like*, which are negative regulatory factors of drought, were significantly down‐regulated in both *GhHUB2‐*knockdown plants and WT plants. The expression levels of *GhMKK2* were higher in *GhHUB2‐*knockdown plants than in WT plants, whether with or without drought treatment. The RT‐qPCR results showed that the expression of *GhDREB*,* GhRD22* and *GhP5CS*, but not *GhPIP2; 7‐like* and *GhMKK2* was related to *AtHUB2* overexpression. These results indicated that *GhHUB2* was involved in the cotton drought stress response; however, the expression of *GhHUB2* was not induced by drought stress, and overexpression of *AtHUB2* was the reason for the improved drought resistance of transgenic cotton.

### AtHUB2 is a functional E3 ligase that increases transgenic cotton histone H2B monoubiquitination through interacting with GhH2B1

**Figure 6:**
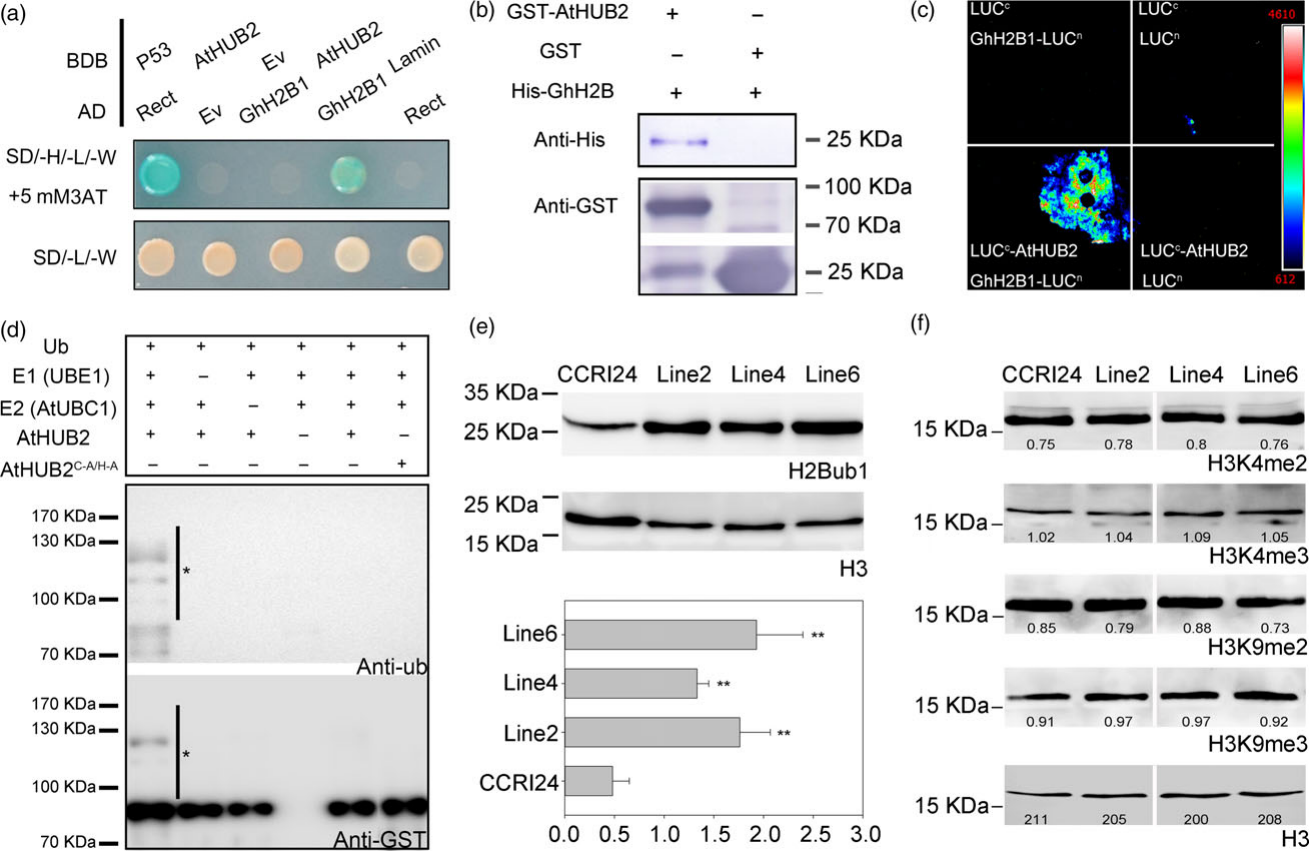
AtHUB2 is a functional E3 ligase. (a) Yeast two‐hybrid (Y2H) assays. GhH2B1‐pGADT7 was transformed into yeast cells with either the pGBKT7 vector alone (BDB) or AtHUB2‐pGBKT7, and growth was monitored on selective medium plates. (b) Pull‐down assay. GST‐HUB2 and GST alone were incubated with GhH2B1‐His. And detected with an anti‐GST antibody and anti‐His antibody. (c) Split‐luciferase assay. GhH2B1‐LUC ^n^ and LUC ^c^‐AtHUB2 co‐injection tobacco. The combinations of LUC ^n^ and LUC ^c^, LUC ^n^ and LUC ^c^‐ AtHUB2, and GhH2B1‐LUC ^n^ and LUC ^c^ were used as negative controls. (d) AtHUB2 possesses E3 ubiquitin ligase activity *in vitro*. GST‐AtHUB2 and the point mutant GST‐HUB2 ^C346A/H348^ were tested for E3 ubiquitin ligase activity. Anti‐Ub and anti‐GST antibodies were used to detect ubiquitinated proteins and GST‐AtHUB2 variants respectively. The asterisk indicates the polyubiquitinated proteins. (e) Global histone modification in the control (CCRI24) and transgenic lines. H2B monoubiquitination was detected with an anti‐H2Bub1 antibody, and H3 was used as a loading control. ImageJ software was used to analyse the greyscale value of each band. Vertical bars represent SD (**P < 0.01). (f) Detection of H3K4 methylation on a global scale in the control (CCRI24) and transgenic lines. And an anti‐H3K4me2 and anti‐H3K4me3 antibodies were used to detect target band, respectively. H3K9me2 and H3K9me3 were used as unchanged controls, and H3 was used as a loading control. The number below the lane indicates the greyscale value of each band.

To understand how *AtHUB2* overexpression improves transgenic cotton drought tolerance, we performed a yeast two‐hybrid assay to screen a cotton whole‐growth‐period cDNA library using AtHUB2‐pDEST32 (At1g55250.2) as bait, and a series of candidate genes were identified (Table S2). The cotton histone GhH2B1, which was previously identified as candidate GhHUB2 interact protein (Feng *et al*., 2018), was included in the group of 17 positive clones. Multiple sequence alignment and phylogenetic tree analysis showed that the GhH2B1 C‐terminus and key functional amino acid sites are conserved (Figure S6) (Hwang *et al*., 2003). And GhH2B1 is most closely related to AtHTB9 (AT3G45980), which was previously reported as an AtHUB2‐interacting protein (Cao *et al*., 2008). We then co‐transformed BD‐AtHUB2 (BD, pGBKT7, Clontech) with AD‐GhH2B1 (AD, pGADT7, Clontech) in AH109 yeast to confirm the interaction between AtHUB2 and GhH2B1 (Figure 6a). The results of *in vitro* pull‐down and split‐luciferase assays led to the same conclusion (Figure 6b,c).

AtHUB2 has a conserved C3HC4 RING‐type domain that controls histone H2B monoubiquitination. We tested the E3 ligase activity of AtHUB2 *in vitro*. A purified GST‐AtHUB2 fusion protein was mixed with ubiquitin (ub), rabbit E1 UBE1, and *Arabidopsis* E2 UBC1, and an AtHUB2 point mutant (C^346^A/H^348^A) was used as a negative control (Figure S2). Immunoblot analysis with anti‐ub and anti‐GST antibodies showed that ubiquitinated proteins were detectable only in the presence of all these components, while no ubiquitination was observed in the presence of HUB2^C346A/H348A^ (Figure 6d). These results suggest that AtHUB2 could be autoubiquitinated in the presence of E1 and E2 enzymes and that the C3HC4 RING domain is essential for the protein ubiquitination ability of AtHUB2. We then examined the H2Bub1 modification pattern in the control cultivar CCRI24 and transgenic plants. An anti‐H2Bub1 antibody detected one clear band in all plants, but the level of H2Bub1 was higher in transgenic plants than in control plants. ImageJ software was used to analyse the greyscale value of each band and further confirmed that the H2Bub1 level was significantly improved in transgenic lines (Figure 6e). These findings indicated that AtHUB2 possesses E3 ubiquitin ligase activity and works directly on cotton histone H2B in *AtHUB2*‐overexpressing cotton.

### The increase in H2Bub1 improves H3K4me3 modification at the *GhDREB* chromatin locus in *AtHUB2* transgenic cotton

Previous studies have shown that H2Bub1 is associated with histone H3K4 methylation (Lee *et al*., 2007). To further explain how the global H2Bub1 level influences drought tolerance in transgenic cotton, we performed an immunoblotting assay to analyse H3K4 methylation levels. Anti‐H3K9me2 and anti‐H3K9me3 antibodies were used as negative controls. As shown in Figure 6f, there were no significant differences in the global H3K4 and H3K9 methylation levels between control and transgenic plants. This finding indicates that changes in the global H2Bub1 level had no effect on the global H3K4 methylation level.

**Figure 7:**
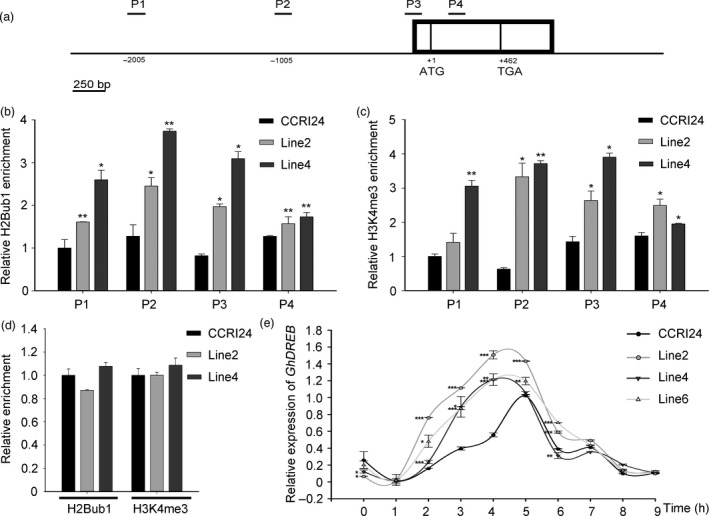
Detection of global histone H2Bub1 and H3K4me3 levels at the *GhDREB* chromatin locus. (a) Schematic diagram of the *GhDREB* gene. The exons are shown in boxes, and the indicated PCR fragments analysed in the ChIP assay are indicated with short black lines. P1: 2000 bp upstream of the ATG; P2: promoter region; P3: transcription start site (TSS) region; P4: gene body region. (b) H2Bub1 at the *GhDREB* locus in the CCRI24 and transgenic lines. Immunoprecipitated DNA was analysed via RT‐qPCR, and enrichment was determined as a percentage of the input. (c) H3K4me3 at the *GhDREB* locus in the CCRI24 and transgenic lines. (d) The *GhUBI* gene was selected as a housekeeping gene that was not differentially expressed in the transgenic lines. (e) Expression of *GhDREB* at different time points of air drought treatment in control and *AtHUB2*‐overexpressing lines. Real‐time RT‐qPCR quantification was normalized to the expression of *GhUBI*. Vertical bars represent SD. (*P < 0.05, **P < 0.01, ***P < 0.001).

The up‐regulated H2Bub1 had no influence on the global H3K4 methylation level, but the expression of drought stress‐related genes was significantly changed in transgenic cotton. Therefore, we examined whether histone trimethylation (H3K4me3) was altered at these drought‐related genes. A ChIP assay was performed with an anti‐H2Bub1 antibody and an anti‐H3K4me3 antibody against chromatin derived from four‐week‐old WT and *AtHUB2*‐overexpressing lines (line2 and line4) respectively. Then, we used RT‐qPCR to detect the enrichment of H2Bub1 and H3K4me3 in the chromatin of these drought‐related genes. The results indicated that H2Bub1 and H3K4me3 enrichment was only obviously detectable at the *GhDREB* chromatin locus, while no or only weak enrichment was detected at the other genes (date not shown). Moreover, a significant increase in the level of H2Bub1 was observed across the *GhDREB* P1‐P4 region in transgenic cotton (Figure 7b). In addition, the H3K4me3 level was increased at the promoter (P2) and transcription start site (TSS) (P3) and in the gene body (P4) of *GhDREB* (Figure 7c), whereas no difference was observed in the *UBI1* chromatin region (Figure 7d). To determine the effect of H2Bub1 and H3K4me3 enrichment at the *GhDREB* chromatin locus, we detected the spatio‐temporal expression of *GhDREB*. As shown in Figure 7e, with a longer air‐drying time, the expression level of *GhDREB* was gradually increased in both WT and *AtHUB2*‐overexpressing plants, but the expression levels were higher in the latter. Additionally, the expression levels reached a maximum at 4 h in *AtHUB2*‐overexpressing plants compared to 5 h in WT. These results indicated that the H2Bub1 was associated with H3K4me3 at the *GhDREB* locus, and the higher H3K4me3 modification would trigger a quick response of *GhDREB* to drought stress in *AtHUB2* transgenic cotton.

## Discussion

AtHUB1/2 are major enzymes responsible for histone H2B monoubiquitination in *Arabidopsis*, which affects many aspects of plant growth and development, including cellular proliferation, seed dormancy, and immune and salt stress responses. This study is the first report showing that *AtHUB2* plays a role in drought stress and obtaining *AtHUB2*‐overexpressing drought‐resistant cotton plants with stable inheritance, as demonstrated by increased plant height, fruit branch numbers and boll numbers under drought conditions.

In plants, H2Bub1 is a marker of active chromatin and is broadly involved in the regulation of gene transcription. Our results showed that the TFs *GhRD22* and *GhDREB* and the proline synthesis‐related gene *GhP5CS* were significantly up‐regulated in transgenic cotton, while these genes were lower in *GhHUB2‐*knockdown plants that encounter drought stress compared to the CCRI24 cultivar (Figure 4). The immunoblotting results showed that *AtHUB2* overexpression increased global H2Bub1 levels, but we detected H2Bub1 enrichment only at the *GhDREB* gene locus. H2Bub1 enrichment at the other genes was almost undetectable. The drought stress response is a complex process involving multiple signalling and regulatory pathways. H2Bub1 may function at upstream genes or through an indirect pathway to influence the expression of these genes, and the rapid response of *GhDREB* after drought stress may provide a signal for other pathways to regulate the expression of the other genes.

The DREB belongs to a large group of plant‐specific TFs known as the APETALA 2/ethylene‐responsive element‐binding factor (AP2/ERF) family. In *Arabidopsis,* there are eight *DREB2s*, and *DREB2A* and *DREB2B* are highly induced by drought stress (Mizoi *et al*., 2012). However, their functions under drought stress remain unclear. There is a negative regulatory domain (NRD) in DREB2 that keeps the protein inactive under normal conditions. The activity of DREB2 can be modulated by posttranslational regulatory mechanisms, including ubiquitination, phosphorylation and alternative splicing (Agarwal *et al*., 2007; Matsukura *et al*., 2010; Qin *et al*., 2008). In *Arabidopsis,* the DREB2A‐interacting protein DBP3‐1 was found to form a transcriptional complex that contributes to the target gene selectivity of DREB2 under heat stress (Sato *et al*., 2014). In addition to posttranslational regulation, epigenetic modifications also participate in the transcriptional regulation of *DREB* family genes. Under osmotic stress, the levels of H3K9 and H4K5 acetylation in the *ZmDREB2A* promoter region increase quickly to activate *ZmDREB2A* expression (Zhao *et al*., 2014). Differential acetylation of histone H3 at the promoter can facilitate the activation of *OsDREB1b* transcription (Roy *et al*., 2014). According to ChIP‐Seq data published in 2010, 90% of annotated *Arabidopsis* genes carry H3K4 methylation marks; and H3K4me3 was broadly distribute on the nucleosomes of stress‐induced genes, including *DREB2* (van Dijk *et al*., 2010). Our results further indicate that the epigenetic modifications participate in *DREB* gene transcriptional control in cotton. The *GhDREB* chromatin locus was enriched with H2Bub1 and H3K4me3 modifications. Increased H2Bub1 could elevate the H3K4me3 modification at the *GhDREB* chromatin locus in *AtHUB2* transgenic cotton. And decreased H2Bub1 would reduce the H3K4me3 modification at the *GhDREB* chromatin locus in *GhHUB2‐*konckdown transgenic cotton (Figure S7). H3K4me3 always linked to the transcriptional activation of related genes. As show in Figures 7 and S7, elevated H3K4me3 promoted *GhDREB* expression, and when the H3K4me3 was impaired, the expression of *GhDREB* was lower than control. Hyper‐methylation of H3K4 is a mark of ‘stress memory’, as previous exposure to abiotic stress can make plants more resistant to future exposure, which is accompanied by high levels of H3K4me3 on nucleosomes (Ding *et al*., 2012; Liu *et al*., 2014). Our results demonstrated that the levels of H3K4me3 at the *GhDREB* chromatin locus were higher in *AtHUB2* transgenic cotton. When the transgenic cotton encountered drought stress, the expression level of *GhDREB* was quickly up‐regulated because of ‘stress memory’ (Figure 7e).

The expression of a series of drought‐related genes is under the control of *DREB*, which makes it a useful tool for breeding stress‐resistant transgenic plants. However, the direct overexpression of *DREBs* in transgenic *Arabidopsis* leads to severe growth retardation. However, in our study, the overexpression of *AtHUB2* in transgenic cotton changed the epigenetic modifications at the *GhDREB* chromatin locus, allowing a lower expression level under normal growth conditions without any other effects on plant development. When the transgenic cotton encounters drought stress, the increased H3K4me3 modification at the *GhDREB* chromatin locus allows it to quickly respond to drought stress to improve transgenic plant drought resistance. Our results provide useful tools for breeding drought‐resistant transgenic plants.

## Experimental procedures

### Plant materials

The full‐length *AtHUB2* (NCBI accession number: BT029207) CDS was cloned into the pCambia1305.1 vector with a Myc tag added to its 5′ end, and the expression of the Myc‐AtHUB2 fusion protein was driven by the cauliflower mosaic virus (CaMV) 35S promoter. The *AtHUB2* overexpression vector was transformed into the cotton cultivar CCRI24 via *Agrobacterium*‐mediated transformation, and 12 lines of T0 transgenic plants were obtained. The T0 plants were used to generate T1 progeny via self‐fertilization, and the presence and integrity of the transgene were confirmed by PCR analysis of genomic DNA with specific primers (Table S3). The AtHUB2 protein was detected with anti‐Myc antibody (Sigma).

Seeds of the wild‐type (WT) *Arabidopsis* line Columbia‐0 (Col‐0), the *hub2‐2* (Salk_071289) mutant line and the *hub2‐2* mutant line complemented with *HUB2* (*HUB2/hub2‐2*) were used in the experiments (Col‐0 background). The T‐DNA insertion mutant of *hub2‐2* (Salk_071289) was obtained from the ABRC (Ohio State University, Columbus, OH). The complemented line *HUB2/hub2‐2* was produced by transforming *hub2‐2* mutant plants with the pCAMBIA1200 vector expressing *HUB2* genomic DNA (including both introns and exons) driven by the CaMV 35S promoter.

### Plant growth conditions and drought treatment

Cotton seeds were placed on wet germination paper, incubated at 30 °C for 2 days for germination and then planted in 12*12*15 cm^3^ pots containing 1 L of soil mixture. These plants (≥30 for each line) were grown in a greenhouse at 28 ± 2 °C with a 16/8 h photoperiod for 28 days and then divided into two groups: a well‐watered group, as a control, and a drought treatment group. For drought treatment, water was withheld for 35 days, and photographs were taken on days 20, 28 and 35. The plants were then re‐watered for 1 week for recovery.

Field drought treatments were carried out in Anyang, Henan province, China. The control plants (cultivar CCRI24) and the homozygous transgenic cotton lines were divided into two groups. One group was well‐watered as a control, while the second group was grown in a waterproof shed. The waterproof shed was irrigated with 30 tons/acre water 1 week before sowing. When the soil water content was 16%–20%, the seeds were sown. The soil water content was then detected every 30 days, and re‐watering was performed after 3 months of drought treatment (35 tons/acre). The photographs were taken 1 month after re‐watering.

Seeds of *Arabidopsis* WT (Col‐0) plants, the *hub2‐2* (Salk_071289) mutants and *hub2* mutant complemented with *HUB2* (*HUB2/hub2‐2*) were sterilized and incubated for 3 days at 4 °C in the dark. For seedling assays, sterile seeds were germinated on half‐strength MS medium for 3 days and then transferred to half‐strength MS agar medium infused with different concentrations of polyethylene glycol 8000 (PEG‐8000) (Verslues and Bray, 2004). The plates were incubated at 22 °C with a 16/8 h photoperiod. For soil dehydration assays, plants were germinated on half‐strength MS medium, grown in soil at 22 °C with a 16 h light photoperiod for 14 days and then grown under the same conditions with or without additional water for 12 days. Photographs were taken before drought treatment and on day 12 after drought treatment and re‐watered for 3 days.

### Southern blotting

Genomic DNA was extracted from 4‐week‐old CCRI24 and *AtHUB2* transgenic cotton plants according to Paterson's method (Paterson *et al*., 1993). Fifty micrograms of genomic DNA was digested with the *Bam*HI restriction enzyme (NEB), separated on a 0.8% agarose gel, and transferred onto a Hybond‐N^+^ nylon membrane (Amersham) by capillary blotting. Hybridization was performed according to the DIG High Prime DNA Labeling and Detection Starter Kit II (Roche).

### Quantitative real‐time PCR analysis

Total RNA was extracted from the leaves of cotton plants grown under normal conditions or plants subjected to drought treatment using an RNAprep Pure total RNA extraction kit for polysaccharide‐ and polyphenol‐rich plants (TIANGEN), and first‐strand cDNA synthesis was performed using 2.0 μg of total RNA, an Oligo‐dT (18) primer and M‐MLV reverse transcriptase (Promega). Quantitative real‐time RT‐PCR (RT‐qPCR) analysis was performed in a CFX‐96 real‐time system (Bio‐Rad) using SYBR Premix Ex Taq (TaKaRa). The expression levels of the genes mentioned above were examined using specific primers. *GhUBI1* was used as an internal control (Wang *et al*., 2013). All the primers used in this study are shown in Table S3.

### Measurement of proline, soluble sugars and relative water content (RWC)

Leaves from normal control or stress‐treated plants at similar developmental stages were used to measure proline content, soluble sugar content and RWC. Proline was measured according to the method of Bates (Bates *et al*., 1973). Soluble sugars were measured using the anthrone reagent (Dubois *et al*., 1956). The RWC of leaves was assayed as described by Parida (Parida *et al*., 2007).

### Yeast two‐hybrid assays

A cotton whole‐growth‐period two‐hybrid library was constructed by Invitrogen (Shanghai, China). RNA was extracted from cotton at four stages of development. The pDEST22 vector system was then constructed. The bait gene was inserted into the pDEST32 vector via the Gateway^**®**^ recombination reaction, and the two‐hybrid library was screened according to the protocol for the ProQuest^TM^ two‐hybrid system provided by Invitrogen.

To test specific two‐hybrid interactions, AtHUB2 was inserted into the pGBKT7 vector as bait, and the interaction partners were inserted into the pGADT7 vector as prey. The bait and prey constructs were co‐transformed into yeast AH109 cells. Positive clones were identified by the ability to grow on SD medium lacking histidine/leucine/tryptophan (SD/‐His/‐Leu/‐Try) and containing 5 mM 3‐aminotriazole (3AT).

### Split‐luciferase and pull‐down assays

*AtHUB2* and *GhH2B1* were fused to the C‐ and N‐termini of the firefly LUC enzyme to produce LUC^c^‐AtHUB2 and GhH2B1‐LUC^n^ respectively. LUC^c^ and LUC^n^ were used as negative controls. LUC^c^‐AtHUB2, GhH2B1‐LUC^n^, LUC^c^ and LUC^n^ were transiently expressed in *Nicotiana benthamiana*. Leaves were collected 3 days after infiltration and infiltrated with 1 mm luciferin before being observed. Fluorescence was detected using a Tanon 5200 chemiluminescence imaging system (Tanon).

Recombinant (glutathione S‐transferase) GST‐AtHUB2 and GhH2B‐His (histidine) containing plasmids were transformed into *Escherichia coli* BL21 (DE3) cells, and expression was induced with 0.5 mm or 1 mm isopropyl β‐D‐1‐thiogalactopyranoside (IPTG). Recombinant fusion proteins were affinity purified from bacterial lysates according to the GE protocol (www.gelifesciences.com/protein-purification). Purified GST‐AtHUB2 and GhH2B‐His were used for *in vitro* binding experiments according to a previously described protocol (Xie *et al*., 1999).

### *In vitro* ubiquitination and immunoblotting assays

Rabbit E1 (Boston Biochem, 200 ng), purified *Arabidopsis* E2 AtUBC1‐His (500 ng), purified GST‐AtHUB2 (1 mg) and recombinant plant ubiquitin (Boston Biochem, 80 μg) were used for the *in vitro* ubiquitination assay. An AtHUB2 protein carrying two point mutations (C^345^A & H^347^A) was used as the negative control for fully functional AtHUB2. Reactions were performed at 37 °C for 2 h and then stopped by the addition of an equal volume of loading buffer (40 mm Tris‐HCl, pH 6.8, 2% SDS, 10% glycerol, 0.1% Coomassie Brilliant Blue, 0.1% bromophenol blue and 1% β‐mercaptoethanol) and boiling for 10 min.

For total plant protein extraction, leaves were ground in liquid nitrogen and dissolved in denaturing buffer containing 50 mm Tris‐HCl, pH 7.5, 150 mm NaCl, 0.1% NP‐40, 4 m carbamide and 1 mm PMSF. Protein concentrations were determined using a Coomassie protein assay kit (Bradford). Each total protein sample was analysed by 10% SDS‐PAGE and immunoblotting using relevant antibodies. The specific antibodies employed in these assays were as follows: anti‐H3 (Cell Signaling Technology); anti‐H3K4me2 (Millipore); anti‐H3K4me3 (Millipore); anti‐H3K9me2 (Millipore); anti‐H3K9me3 (Millipore); anti‐ub (abcam); and anti‐GST (Cell Signaling Technology).

### ChIP assay

ChIP experiments were performed as previously described (Saleh *et al*., 2008) with some modifications. A 3 g sample of leaves was fixed with cross‐linking buffer and 1% formaldehyde using vacuum infiltration two times for 15 min each, and the cross‐linking reaction was quenched with 0.125 m glycine. The leaves were ground in a mortar and pestle in liquid nitrogen, resuspended in nuclei isolation buffer and then filtered through Miracloth. After centrifugation, the pellets (nuclei) were resuspended in cold nuclei lysis buffer and sonicated (Bioruptor^®^ Plus sonication device, Diagenode, Belgium). After centrifugation, the supernatant was pre‐cleared with Magna ChIP™ Protein A Magnetic Beads (EMD Millipore corporation, Temecula, CA), and specific antibodies were added and incubated overnight at 4 °C. The specific antibodies used were as follows: anti‐H2Bub1 (Cell Signaling Technology, Danvers, MA) and anti‐trimethyl‐H3K4 (Abcam). The enriched DNA fragments were detected by RT‐qPCR and compared with the input samples, the amount of immunoprecipitated chromatin as normalized to the total amount of chromatin used in GhDREB P1 region in wild‐type plants was given as 1. *GhUBI1* was used as a negative control.

### Phylogenetic analysis

The phylogenetic tree was constructed from the indicated amino acid sequences using the MEGA5 programme based on the neighbor‐joining method with 1000 bootstraps. The numbers beside the branches represent bootstrap values based on 1000 replicates. Sequences were obtained from the National Center for Biotechnology Information (NCBI) and The *Arabidopsis* Information Resource (TAIR) databases. The multiple sequence alignment of the indicated sequences was analysed with CLUSTAL W2 or CLC Sequence Viewer 7.0.

### Statistical analysis

All experimental data are the means of at least three independent replicates, and statistical analyses were performed using Microsoft Office Excel 2007, Microsoft Corporation, Redmond, WA. Variations among transgenic and control plants subjected to different treatments were compared using one‐way analysis of variance (ANOVA). (*P < 0.05; **P < 0.01; ***P < 0.001).

## Authors’ contributions

J‐L.D. and T.W.: led the study and revised the manuscript. H.C.: performed the main experiments and wrote the manuscript. H.F. participated in vector construction. X‐Y.Z.: participated in low‐generation detection and purification of transgenic materials. C‐J.Z.: participated in plant material preparation. All authors read and approved the final manuscript.

## Conflict of interest

The authors declare no competing financial interests.

## Supporting information


**Figure S1** Response of *Athub2* mutants to dehydration stress.
**Figure S2** Characteristics of *AtHUB2*.
**Figure S3** Generation and molecular characterization of transgenic cotton lines expressing *AtHUB2*.
**Figure S4** Agronomic traits of different *AtHUB2* transgenic lines in the field.
**Figure S5** *AtHUB2* significantly enhances the drought tolerance of transgenic cotton in the greenhouse.
**Figure S6** Multiple sequence alignment and phylogenetic tree analysis of GhH2B1 with *Arabidopsis* histone H2Bs.
**Figure S7** Detection *GhDREB* chromatin locus H2Bub1 and H3K4me3 levels in *GhHUB2‐*knockdown plants.
**Table S1** Comparison of drought‐related indexes between transgenic cotton lines and controls.
**Table S2** Positive clones from library screening.
**Table S3** Primers used in this study.
