# Supplementary material for: An Arabidopsis E3 ligase HUB2 increases histone H2B monoubiquitination and enhances drought tolerance in transgenic cotton
Source: Plant Biotechnol J. 2018 Sep 4;17(3):556–68. doi: 10.1111/pbi.12998 (PMC6381789; doi:10.1111/pbi.12998)
Supplement: Supplementary file 1 — Figure S1 Response of Athub2 mutants to dehydration stress. Figure S2 Characteristics of AtHUB2. Figure S3 Generation and molecular characterization of transgenic cotton lines expressing AtHUB2. Figure S4 Agronomic traits of different AtHUB2 transgenic lines in the field. Figure S5 AtHUB2 significantly enhances the drought tolerance of transgenic cotton in the greenhouse. Figure S6 Multiple sequence alignment and phylogenetic tree analysis of GhH2B1 with Arabidopsis histone H2Bs. Figure S7 Detection GhDREB chromatin locus H2Bub1 and H3K4me3 levels in GhHUB2‐knockdown plants. Table S1 Comparison of drought‐related indexes between transgenic cotton lines and controls. Table S2 Positive clones from library screening. Table S3 Primers used in this study. [file PBI-17-556-s001.pdf]

## Supporting Information

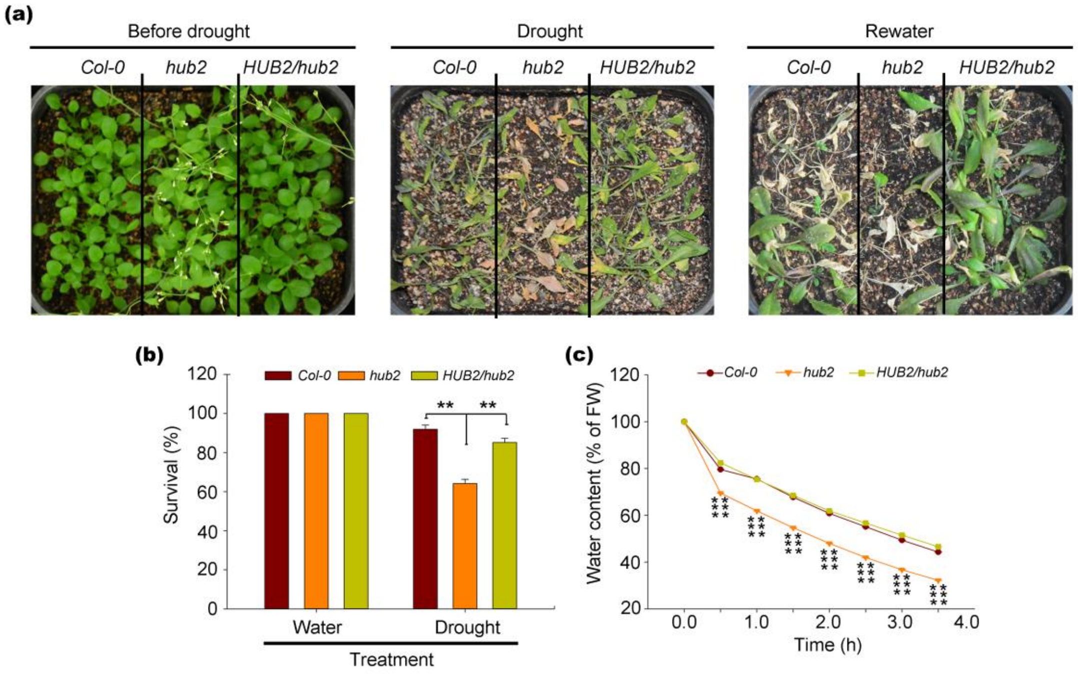


**Figure S1.** Response of *Athub2* mutants to dehydration stress. (a) Drought sensitivity of wild-type (WT), *hub2* mutant and *HUB2/hub2* complementation line seedlings. At 2 weeks after germination (Before drought), the plants were grown without water (Drought) for 12 days, followed by 3 days of watering for recovery (Rewater). (b) Survival percentages of the genotypes indicated in (a) after 12 days of drought treatment, followed by the 3-day recovery period. (c) The volume of water lost via air-drying from detached leaves of the indicated genotypes (4 weeks old) was measured as the percentage of change in the fresh weight (FW) of the leaves. Vertical bars represent the SD from three independent experiments ($n \geq 30$) (**$P < 0.01$).

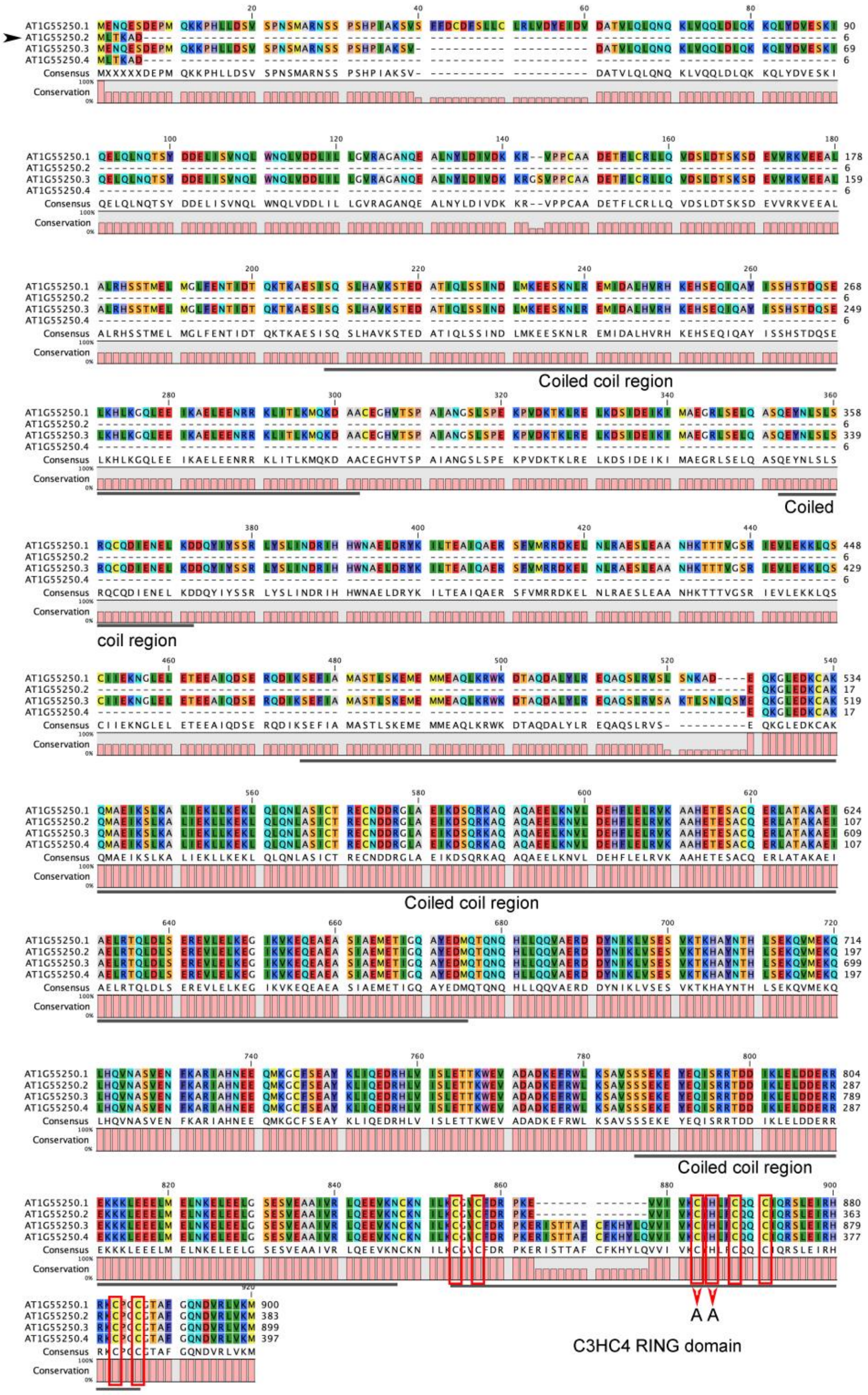


**Figure S2.** Characteristics of *AtHUB2*. Multiple sequence alignment of the four *AtHUB2* alternative splicing isoforms (AT1G55250.1, AT1G55250.2, AT1G55250.3, and AT1G55250.4). The black arrow indicates *AtHUB2* cloned by our group. The red arrow indicates key functional sites in the conserved C3HC4 RING domain.

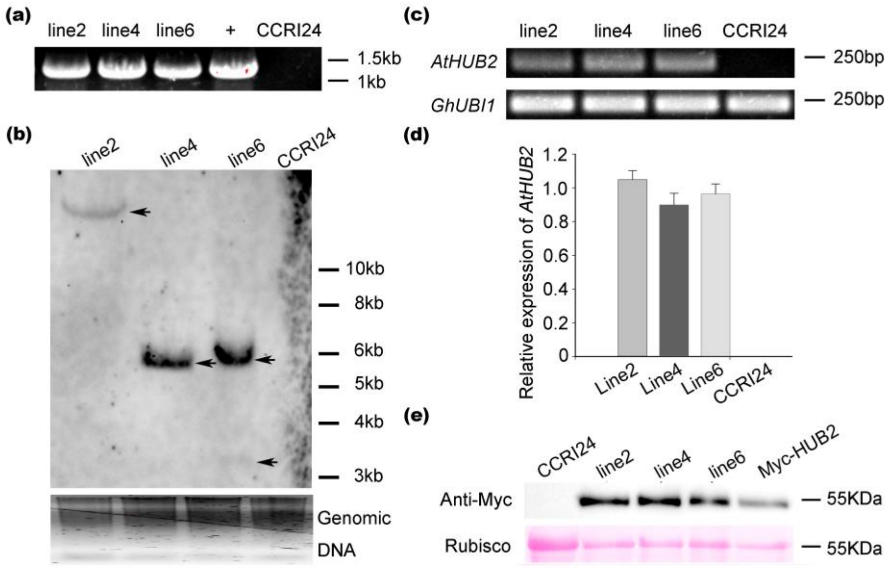


**Figure S3.** Generation and molecular characterization of transgenic cotton lines expressing *AtHUB2.* (a) PCR analysis of *AtHUB2* in the genomic DNA of plants from the T2 generation. (b) Southern blotting analysis-positive *AtHUB2* transgenic cotton plants; the arrow indicates the target strip. The genomic DNA was as loading control. (c) Semi-quantitative RT-PCR analysis of *AtHUB2* expression in three T2 transgenic cotton lines. (d) RT-qPCR analysis of *AtHUB2* expression in three T2 transgenic cotton lines. (e) Immunoblotting analysis of the expression of the AtHUB2 protein in three T2 transgenic cotton lines. An anti-Myc antibody was used to detect the AtHUB2 protein, Myc-HUB2 was used as the positive control, and rubisco protein was as a loading control. The black line indicates the strip size.

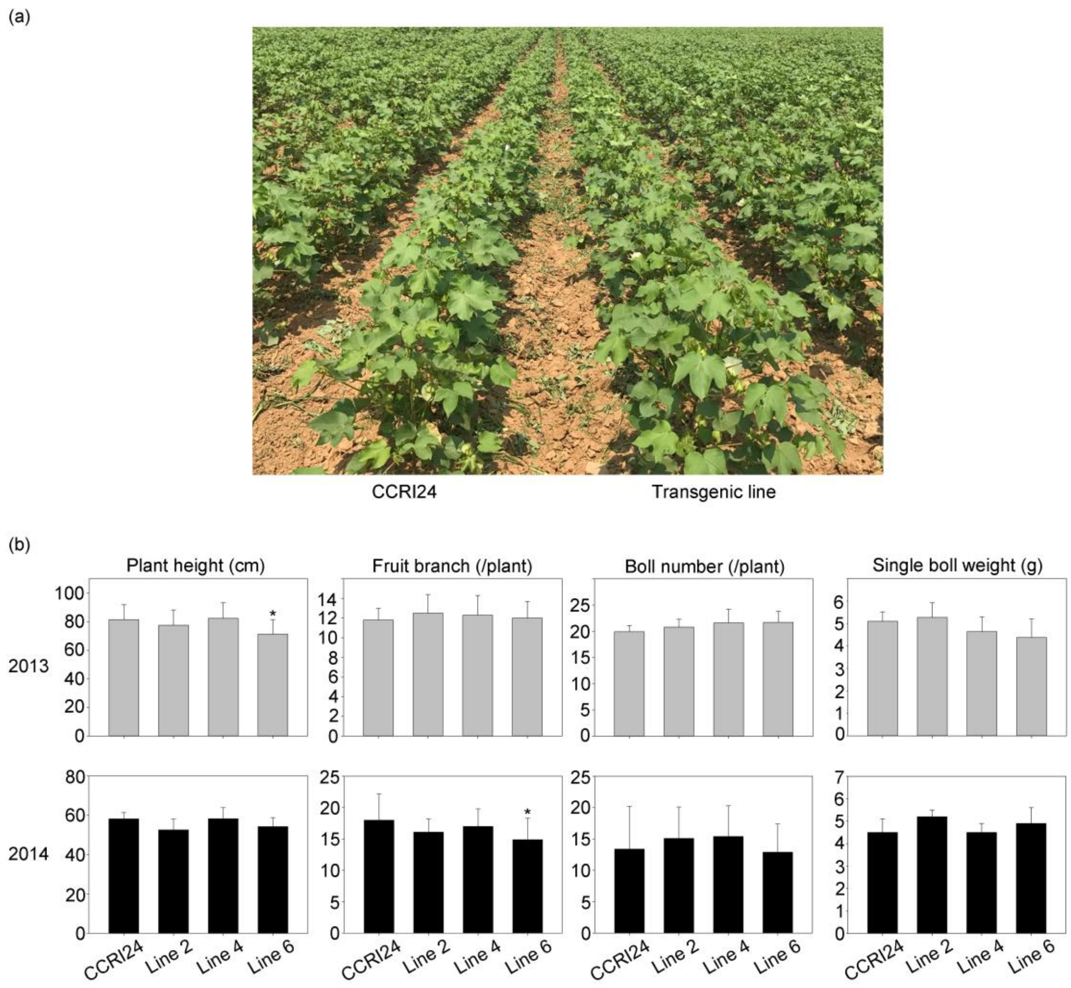


**Figure S4.** Agronomic traits of different AtHUB2 transgenic lines in the field. (a) Wild-type (WT) and *AtHUB2*-expressing cotton lines were grown under normal conditions. Photographs were taken 90 days after germination. (b) Plant height, fruit branching, boll number, and single boll weight of the control (CCRI24) and three independent transgenic lines (line2, line4, and line6) grown in the field ($n \geq 30$). Vertical bars represented the SD. (*$P < 0.05$).

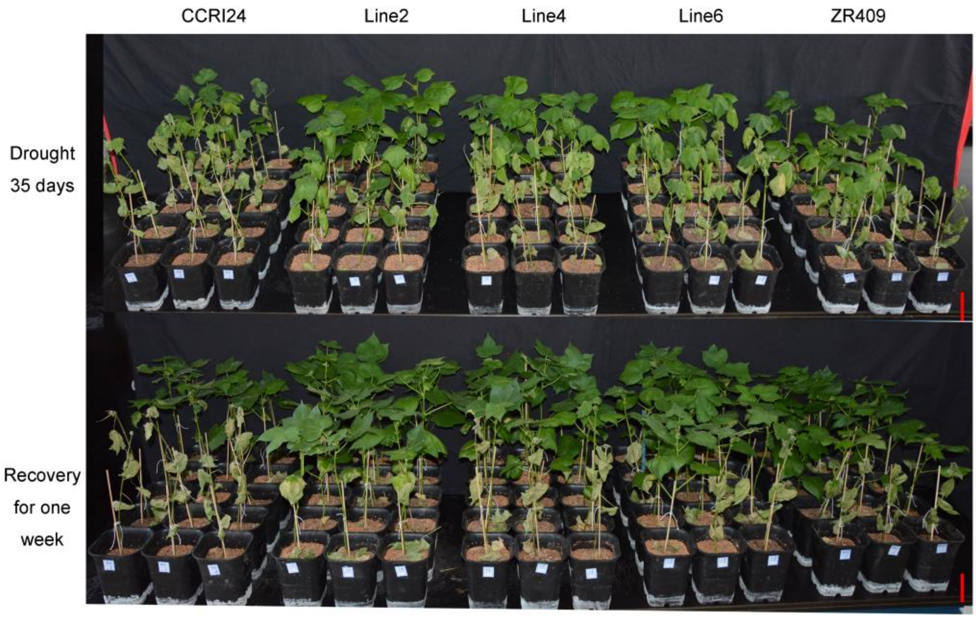


**Figure S5** *AtHUB2* significantly enhances the drought tolerance of transgenic cotton in the greenhouse. Drought tolerance of control plants and *AtHUB2*-expressing cotton plants. Photographs were taken after 35 days of drought stress and after a 1-week recovery period. Bar = 7.5 cm.

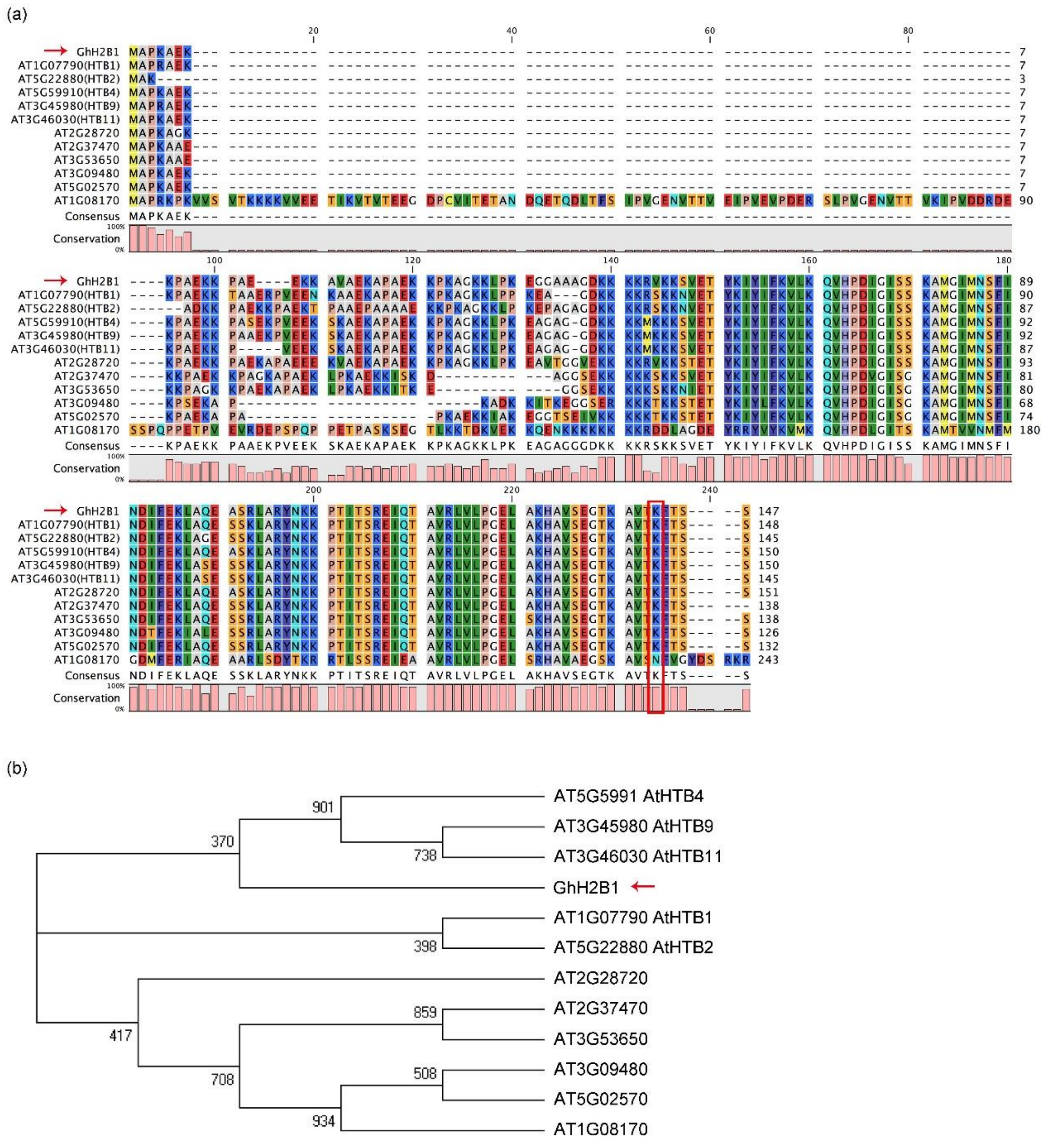


**Figure S6** Multiple sequence alignment and phylogenetic tree analysis of GhH2B1 with *Arabidopsis* histone H2Bs. (a) Multiple sequence alignment was performed using CLC Sequence Viewer 7.0, and (b) the phylogenetic tree was constructed with the MEGA5 programme using the neighbour-joining method with 1,000 bootstraps. The red arrow indicates cotton histone H2B (GhH2B1).

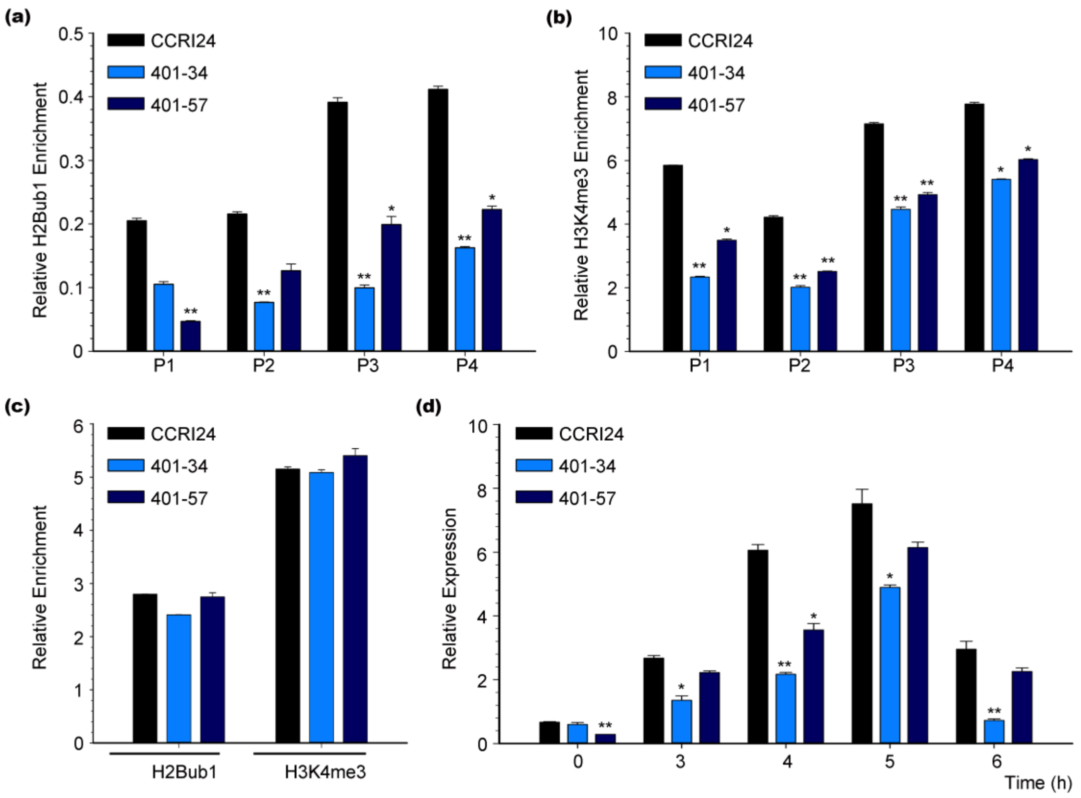


**Figure S7** Detection *GhDREB* chromatin locus H2Bub1 and H3K4me3 levels in *GhHUB2*-knockdown plants. (a) H2Bub1 at the *GhDREB* locus in the CCRI24 and *GhHUB2*-underexpression plants. (b) H3K4me3 at the *GhDREB* locus in the CCRI24 and transgenic lines. (c) The *GhUBI* gene was selected as a housekeeping gene that was not differentially expressed in the transgenic lines. (d) Expression of *GhDREB* at different time points of air drought treatment in control and *GhHUB2*-knockdown lines. Real-time RT-qPCR quantification was normalized to the expression of *GhUBI*. Vertical bars represent SD. (*P < 0.05, **P < 0.01).

**Table S1. The drought related indexes between transgenic cotton lines and controls.**

| NO. | wilting | chlorosis | lodging |
|---|---|---|---|
| CCRI24 | 2 | 1 | 1 |
| Line 2 | 1 | 0 | 0 |
| Line 4 | 1 | 1 | 0 |
| Line 6 | 1 | 1 | 0 |
| ZR409 | 1 | 0 | 0 |

Wilting 1: part of the leaves mild wilting; 2: all of leaves mild wilting; 3: part of leaves mild wilting part severe wilting; 4 most of leaves severe wilting; 5 all of leaves severe wilting

Chlorosis 1: part of the leaves mild chlorosis; 2:1/3 leaves chlorosis; 3: part of leaves yellow; 4: part of leaves fall off; 5: leaves withered

Lodging 1: angle of inclination < 30°; 2: part angle of inclination >45°; 3: almost angle of inclination >45°; 4: part lodging; 5: all lodging

**Table S2. Positive Clones from Library Screening.**

| NO. | Blast description | Accession | Repeats |
|---|---|---|---|
| 1 | Histone H2B1 [Gossypium hirsutum] | AAB97163.1 | 1 |
| 2 | SINAH1 protein [Gossypium hirsutum] | AAD53877.1 | 2 |
| 3 | RING/U-box superfamily protein isoform 1 [Theobroma cacao] | XP_007029018.1 | 3 |
| 4 | Xyloglucan endotransglucosylase/hydrolase 2 [Theobroma cacao] | XP_007049742.1 | 3 |
| 5 | Uncharacterized protein TCM_014645 [Theobroma cacao] | XP_007037985.1 | 2 |
| 6 | hypothetical protein JCGZ_11289 [Jatropha curcas] | KDP30913.1 | 2 |
| 7 | Histone ubiquitination proteins group [Theobroma cacao] | XP_007052228.1 | 4 |
| 8 | Zinc transporter 11 precursor isoform 1 [Theobroma cacao] | XP_007020535.1 | 1 |
| 9 | hypothetical protein CISIN_1g009353mg [Citrus sinensis] | KDO77403.1 | 1 |
| 10 | PREDICTED: ubiquitin-60S ribosomal protein L40-like [Nelumbo nucifera] | XP_010271616.1 | 1 |
| 11 | GIA/RGA-like gibberellin response modulator [Gossypium hirsutum] | AAY28970.1 | 1 |
| 12 | putative adenine nucleotide translocase [Castanea sativa] | AAL15894.1 | 1 |
| 13 | Cell division protein ftsH [Populus trichocarpa] | XP_002301927.1 | 1 |
| 14 | heat shock protein 70 [Gossypium hirsutum] | ACJ11745.1 | 1 |
| 15 | uncharacterized protein LOC100306699 [Glycine max] | NP_001235125.1 | 1 |
| 16 | chloroplast chlorophyll A-B binding protein [Gossypium hirsutum] | ACO51067.1 | 1 |
| 17 | Chromatin remodeling 24 [Theobroma cacao] | XP_007048407.1 | 1 |

**Supplemental Table 3. Primers used in this study.**

**For cloning**

| Name | | Primer sequences (5'--3') |
|---|---|---|
| GFP-HUB2 | Forward | ATGGATCCATGCTAACAAAGGCAG |
| | Reverse | CGGTCGACTTACATTTTGACAAGC |
| LUC$^c$-HUB2 | Forward | CCGGGGATCCGATGCTAACAAAGGCAGACGAACAG |
| | Reverse | AATTGTCGACTTACATTTTGACAAGCCGGACG |
| HUB2-GST | Forward | CGCGGATCCATGCTAACAAAGGCAGACGAACAG |
| | Reverse | CCCTCGAGTTACATTTTGACAAGCCGGACGTC |
| HUB2-BD | Forward | CGCCATATGCTAACAA AGGCAGACGA |
| | Reverse | CGCGGATCCTTACATTTTGACAAGCCGGACG |
| HUB2(1-160)-BD | Forward | CGCCATATGCTAACAA AGGCAGACGA |
| | Reverse | CGCGGATCCCAACAAATGT TGGTTCTGTG |
| HUB2(1-260)-BD | Forward | CGCCATATGCTAACAA AGGCAGACGA |
| | Reverse | CGCGGATCCGGCCGACTTG AGCCATCTGA |
| HUB2(160-330)-BD | Forward | CGCCATATGCAGCAGGTGG CTGAGCGGGA |
| | Reverse | CGCGGATCCTTTGAGGATGTTTTTGCAATTC |
| HUB2(260-330)-BD | Forward | CGCCATATGGTGTCTTCAT CCGAAAAGGA |
| | Reverse | CGCGGATCCTTTGAGGATGTTTTTGCAATTC |
| HUB2(330-383)-BD | Forward | CGCCATATG TGTGGTGTAT GTTTTGATCG |
| | Reverse | CGCGGATCCTTACATTTTGACAAGCCGGACG |
| HUB2-pENTR2b | Forward | AGGATCCGATGCTAACAAAGGCAG |
| | Reverse | ACCTCGAGTTACATTTTGACAAGC |
| HUB2-pDEST32 | Forward | GGGGACAAGTTTGTACAAAAAAGCAGGCTTGATGCTA ACAAAGGCAG |
| | Reverse | GGGGACCACTTTGTACAAGAAAGCTGGGTTTACATTT TGACAAGC |
| HUB2 mutant | Forward | GTGGTAATTGTGAAAGCTTATGCCCTG |

| | | |
|---|---|---|
| | Reverse | CTGGCAGAACAGGGCATAAGCTTT |
| pC1305.1-HUB2 | Forward | ATCCATGGTAGAACAGAAGTTGATTTCCGAAGAAGAC CTCATGCTAACA AAGGCAGACG |
| | Reverse | AAACTAGTGTTACATTTTGACAAGCCGGAC |
| CaMV 35S promoter | Forward | GCTCCTACAAATGCCATCA |
| HUB2 | Reverse | GTTACATTTTGACAAGCCGGAC |
| GFP-GhH2B1 | Forward | GGATCCATGGCACCAAAAGCTGAAAA |
| | Reverse | GTCGACTCAAGATGAA GTAAACTTGG |
| GhH2B1-LUC$^n$ | Forward | AATTGAGCTCATGGCACCAAAAGCTGAAAAAAA |
| | Reverse | TTAAGTCGACAAAAGATGAAGTAAACTTGGTCAC |
| GhH2B1-His | Forward | CGCGGATCCATGGCACCAAAAGCTGAAAA AAA |
| | Reverse | CCCTCGAGAAAAGATGAAGTAAACTTGGTCAC |
| GhH2B1-AD | Forward | CCGGAATTCATGGCACCAA AAGCTGAAAA AAA |
| | Reverse | CGCGGATCCTCAAGATGAAGTAAACTTGGTCAC |
| GFP-AtH2B | Forward | GGATCCATGGCGCCGA GAGCAGAGAA |
| | Reverse | GTCGACTCAAGAGCTT GTGAATTTGG |
| AtH2B-LUC$^n$ | Forward | CCGGGGATCCGATGGCGCCGAGAGCAGAGAAGAAG |
| | Reverse | TTAAGTCGACTTTAGAGCTTGTGAATTTGGTAA |
| AtH2B-His | Forward | CGCGGATCCATGGCGCCGAGAGCAGAGAA GAAG |
| | Reverse | CCCTCGAGTTTAGAGCTTGTGAATTTGGTAA |
| AtH2B-AD | Forward | CCGGAATTCATGGCGCCGAGAGCAGAGAA GAAG |
| | Reverse | CGCGGATCCTCAAGAGCTTGTGAATTTGGTAA |
| AtHUB2 southern blot | Forward | CCGAAGAAGACCTCATGCTAACAAAGGCAGAC |
| | Reverse | GATGAAGACACGGCCGACTTGAGCCATC |

**For real-time PCR**

| | | |
|---|---|---|
| AtHUB2 | Forward | GAAGCTGCAATTGTGAGGCTC |
| | Reverse | ATTTCCGGTGTCGGATCTCT |
| GhUBI1 | Forward | CTGAATCTTCGCTTTCACGTTA |

| | | |
|---|---|---|
| | Reverse | GGGATGCAAATCTTCGTGAAA |
| GhP5CS | Forward | AGATGGATTCTGAAAGGAAG |
| | Reverse | CCTTTGACGACCAAGACCA |
| GhPP2C | Forward | ACGCGGTTCAAAACTCAAAA |
| | Reverse | TCCATATCTCGTCTCCTTCCA |
| GhRD22 | Forward | AACAAACCCCAATGCAAAAG |
| | Reverse | AGCACCCTCTAAAGGAACCA |
| GhDREB | Forward | GTTGATTTCGGGTCGGTTT |
| | Reverse | AAGCCAAATCCTCGACTTCTT |
| GhPIP2;7 | Forward | TACTGCACTGCCGGTATCTC |
| | Reverse | CAAGGCAGTTCCCTTGTTGT |
| GhMKK2 | Forward | TTGGTGTGAGTCGGATCTTG |
| | Reverse | GGCCCAATCACCTTGTCTT |
| XP_016725369.1 | Forward | CGGAGGATGGTTTTATTGCT |
| | Reverse | ACCCCTTTTTGGAACCTTTG |
| XP_016729746.1 | Forward | TCTACTACGGCTACGGCAAG |
| | Reverse | CCTCGTTAAAAAGCCATCCA |
| XP_016722486.1 | Forward | TCGTCTTTGTTTCCGGTTTC |
| | Reverse | TACCCTTCATGCACCCTTTC |
| XP_016700483.1 | Forward | CAAGAAAGGGTGCATGAAGG |
| | Reverse | GTCACCGTACATGGCTTTGG |
| GhDREB P1 | Forward | GTCCAAGCAAGAAATTAGGTCTGTATG |
| | Reverse | GAGCTTCAAATAAAACACACACGCC |
| GhDREB P2 | Forward | CAGCCAAGTGGGTAACTAATACTG |
| | Reverse | GTTTCTGGAAACTATAACTTGTAAGCC |
| GhDREB P3 | Forward | CATTCCACTACCAAAAAGGAAGCCC |
| | Reverse | GGTGGCTAGTTGGCAAGTCAAG |
| GhDREB P4 | Forward | CGGAGAGAAGCGAAAGCTGCA |
| | Reverse | GTAGAAAACGGCTGTGTCATAAGC |
